# A Modified Approach to Induce Predictable Congestive Heart Failure by Volume Overload in Rats

**DOI:** 10.1371/journal.pone.0087531

**Published:** 2014-01-31

**Authors:** Sascha Treskatsch, Aarne Feldheiser, Adrian T. Rosin, Marco Sifringer, Helmut Habazettl, Shaaban A. Mousa, Mehdi Shakibaei, Michael Schäfer, Claudia D. Spies

**Affiliations:** 1 Department of Anesthesiology and Intensive Care Medicine, Campus Charité, Mitte and Campus Virchow-Klinikum, Charité - Universitätsmedizin Berlin, Berlin, Germany; 2 Institute of Physiology, Campus Charité Mitte, Charité - Universitätsmedizin Berlin, Berlin, Germany; 3 Institute of Anatomy, Ludwig-Maximilians-Universität München, München, Germany; Maastricht University, Netherlands

## Abstract

The model of infrarenal aortocaval fistula (ACF) has recently gained new interest in its use to investigate cardiac pathophysiology. Since in previous investigations the development of congestive heart failure (CHF) was inconsistent and started to develop earliest 8–10 weeks after fistula induction using a 18G needle, this project aimed to induce a predictable degree of CHF within a definite time period using a modified approach. An aortocaval fistula was induced in male Wistar rats using a 16G needle as a modification of the former 18G needle-technique described by Garcia and Diebold. Results revealed within 28±2 days of ACF significantly increased heart and lung weight indices in the ACF group accompanied by elevated filling pressure. All hemodynamic parameters derived from a pressure-volume conductance-catheter *in vivo* were significantly altered in the ACF consistent with severe systolic and diastolic left ventricular dysfunction. This was accompanied by systemic neurohumoral activation as demonstrated by elevated rBNP-45 plasma concentrations in every rat of the ACF group. Furthermore, the restriction in overall cardiac function was associated with a β1- and β2-adrenoreceptor mRNA downregulation in the left ventricle. In contrast, β3-adrenoreceptor mRNA was upregulated. Finally, electron microscopy of the left ventricle of rats in the ACF group showed signs of progressive subcellular myocardial fragmentation. In conclusion, the morphometric, hemodynamic and neurohumoral characterization of the modified approach revealed predictable and consistent signs of congestive heart failure within 28±2 days. Therefore, this modified approach might facilitate the examination of various questions specific to CHF and allow for pharmacological interventions to determine pathophysiological pathways.

## Introduction

Despite improvements in the treatment of congestive heart failure (CHF), CHF is still associated with a high mortality. The overall prevalence of CHF is still increasing because of the ageing of the population and the success in prolonging survival in patients suffering cardiac disease [1]. Therefore, the number of patients with CHF scheduled for surgery with a consecutive increase in the perioperative risk, especially in high-risk-procedures, e.g. vascular and cardiothoracic surgery, is growing. However, knowledge about the underlying pathophysiological mechanisms, which promote heart failure in the perioperative course resulting in biventricular volume overload remains scarce. Thus, it would be of advantage to have a reproducible and predictable experimental model of heart failure developing within a brief time period.

CHF is a multicausal state in which the heart is unable to deliver an appropriate amount of substrates and oxygen to the tissues [2]. This has been reflected in several experimental animal models with distinct pathophysiological causes: a) coronary ligation – loss of cardiomyocytes, b) aortic banding – pressure overload, c) salt sensitive hypertension – pressure overload, d) genetic models – loss of cardiomyocytes or pressure/volume overload, e) toxic cardiomyopathies – loss of cardiomyocytes [3] and finally f) infrarenal aortocaval fistula (ACF) – volume overload [4].

The experimental model of volume overload induced by an aortocaval fistula was first published by Holman in 1937 and has been established with slight modifications by Garcia and Diebold since 1990 [5]. However, in previous investigations of ACF induced volume overload, heart failure was inconsistent and started to develop earliest 8–10 weeks after fistula induction using a 18G-needle [4], [6]. In a study by Melenovsky et al. overt signs of CHF became first obvious at approximately 20 weeks after ACF placement in male Wistar rats [7] and were only present in 65% at the 22th week [8].

Therefore, in this project we aimed to induce a predictable degree of CHF in every animal within a definite time period using a modified approach (16G needle) of the previously published 18G needle-technique by Garcia and Diebold [5]. Such a model would facilitate the examination of various questions specific to CHF and would allow for precisely timed pharmacological interventions to determine pathophysiological pathways. 28±2 days after ACF induction, the extent of heart failure was determined morphologically, and hemodynamically by means of an intraventricular pressure-volume conductance catheter. Specific adaptations in BNP plasma concentrations and the expression of the β1-, β2- and β3-adrenoreceptor mRNA expression were examined in this modified ACF model. Finally, electron microscopy should reveal subcellular changes according to CHF.

## Materials and Methods

### Animals

Male Wistar rats, 280–300 g (Harlan Winkelmann, Borchen, Germany), were maintained on standard laboratory rat chow and water ad libitum. The animals were kept on a 12-h light–dark cycle. This study was carried out in strict accordance with the recommendations in the Guide for the Care and Use of Laboratory Animals of the National Institutes of Health. The protocol was approved by the Committee on the Ethics of Animal Experiments of the local care authories (Landesamt für Gesundheit und Soziales, Berlin, Germany) (Permit Number: G 0119/08). All surgery was performed under isoflurane (ACF induction) and tiletamine/zolazepam (hemodynamic measurements) anesthesia, and all efforts were made to minimize suffering. Post surgical analgesia was provided by metamizole (40 mg/kg s.c.).

### Experimental model

The needle-technique to induce an infrarenal aortocaval fistula (ACF) has previously been described by Garcia and Diebold using a 18G needle [5]. Here, we modified this technique using a 16G needle. Briefly, a laparotomy was performed and the aorta was punctured with a 16G disposable needle (Braun, Melsungen, Germany) distal to the renal arteries. Then, the needle was advanced through the aortic wall into the adjacent inferior vena cava. After temporarily compressing the aorta and venous vessels above and below the puncture site, the needle was carefully withdrawn and the aortic puncture site was sealed with a drop of cyanoacrylate glue. Patency of the fistula ACF was visualized by the pulsatile flow of oxygenated blood into the vena cava inferior from the infrarenal aorta [9]. Vessels of sham-operated control animals were also temporarily compressed and glued together, but without any puncture of the aorta.

### Hemodynamic measurements

For hemodynamic measurements the “closed chest” method in spontaneously breathing rats was used as described earlier [10]. All measurements were performed under tiletamine/zolazepam anesthesia (Zoletil®, 10 mg/kg s.c. followed by 50 mg/kg i.m.) 28±2 days after fistula induction [11]. The rats were placed on a heating pad to maintain body temperature. After tracheotomy a PE-50 tubing catheter was inserted via the left jugular vein into the superior vena cava for assessment of central venous pressure. Arterial blood pressure was measured by cannulating the right carotid artery with a micro-tip pressure-volume conductance catheter (Millar®, SPR-838 NR). By further advancing the catheter into the left ventricle intraventricular pressures and volumes were registered. The position of the catheter was optimized aiming for maximal stroke volume (SV). For measurement of the parallel conductance volume 100 µl of 15% saline were injected into the central venous line to determine the correction factor for the blood-LV tissue interface, however, inhomogeneity of the electric field was not corrected. Heart rate was derived from the ECG signal. After completion of cannulation there was a 10–15 minute equilibration period before starting the measurements. All measurements were performed in spontaneously breathing rats without mechanical ventilation. All signals were recorded and analyzed by the PowerLab®-system and software (ADInstruments, Dunedin, New Zealand). After completion of the hemodynamic measurements animals were killed by exsanguination and organs were eviscerated for further determinations.

### Determination of BNP plasma concentration

Blood samples for rat brain natriuretic peptide 45 (rBNP-45) determinations were withdrawn from control (n = 6) and ACF (n = 6) animals into EDTA-preloaded tubes after completion of hemodynamic measurements. The blood was centrifuged at 4°C at 1,000 *g* for 10 min immediately after withdrawal, and the plasma was maintained at −80°C until extraction. Plasma rBNP-45 concentrations were measured by using a sensitive enzyme-linked immunosorbent assay (ELISA) kit (Abnova, Heidelberg, Germany) [12].

### β1-, β2- and β3-adrenoreceptor RT-PCR

Tissue of the left ventricle for determination of β1-, β2- and β3-adrenoreceptor (AR) mRNA was obtained from sham-operated control (n = 6) and ACF (n = 6) animals after completion of hemodynamic measurements. Following mRNA extraction from rat cardiac tissue reverse transcription-polymerase chain reaction (RT-PCR) analysis was performed as described previously [13]. In brief, total RNA was prepared with the commercially available kit Qiazol Lysis Reagent, (Qiagen, Hilden, Germany) according to the manufacturer's protocol. Then, 1 µg RNA determined by Nanodrop (Peqlab, Erlangen, Germany) were used for transcription to cDNA by using the Omniscript RT Kit (Qiagen) as follows: 0.5 mM dNTP, 5 µM random primer, 10 U RNase Inhibitor, and 4 U Omniscript reverse Transcriptase. Samples were incubated at 42°C for 1 hour, and cDNA was stored at −20°C. The following primers were used: 5′-CTGCTACAACGACCCCAAGTG-3′ (forward) and 5′-AACACCCGGAGGTACACGAA-3′ (reverse) for β1-AR [14], 5′-GAGCCACACGGGAATGACA-3′ (forward) and 5′-CCAGGCGATAACCGACATGA-3′ (reverse) for β2-AR [14] and 5′-GCCGAGACTACAGACCATAACCA-3′ (forward) and 5′-CATTACGAGGAGTCCCACTACCA-3′ (reverse) for β3-AR [15]. Taqman qRT-PCR was performed with a SYBR Green kit following the manufacturer's instructions (Applied Biosystems, Foster City, CA). Experiments were repeated three times. β1-, β2- and β3-AR mRNA was quantified by using triplicates of samples with the ABI Prism 7500 sequence detection system (Applied Biosystems, System Software v 1.3.1.) and the delta-delta CT method. The housekeeping gene 18S rRNA (5′-CGGCTACCACATCCAAGGAA-3′ forward and 5′-GCTGGAATTACCGCGGCT-3′ reverse) was used as an internal reference gene.

### Electron microscopy

Rats of each group were anesthetized with tiletamine/zolazepam (Zoletil®) (see above) and transcardially perfused with 100 ml warm saline, followed by 300 ml 4% paraformaldehyde/0.1% glutaraldehyde/0.2% picric acid solution (pH 7.4) in 0.16 M phosphate buffer solution (pH 7.4). After perfusion the hearts were removed and postfixed for 3 hours at 4°C in the fixative solution. Tissue of the left ventricle was processed for electron microscopy as described previously [16].

### Statistical analysis

All tests were performed using SPSS 20.0 software program. Normal distribution was analyzed with the Kolmogorov-Smirnov test. Results are expressed as means ± SEM or median and interquartile distance. Statistical significance between the two groups was analyzed by Student t-test or Mann-Whitney test as appropriate. P<0.05 was considered statistically significant.

## Results

### Increased heart and lung weight indices in rats with aortocaval fistula

Values for organ weight, body weight, and the organ weight/body weight ratio (“index”) are given in Table 1. Body weights were not significantly different between the control and the ACF group 28±2 days after fistula induction. However, heart and lung weight indices were significantly increased (p<0.01) at 28±2 days after induction of the aortocaval fistula. Considering technical aspects of the here presented approach, the main complication was uncontrollable bleeding at the puncture site at ACF induction. However, after a learning phase (approximately 30 animals) which resulted in proper needle handling, i.e a) careful puncture of the anterior aortic wall not injuring the posterior wall, and b) very gentle advancement of the needle into the vena cava inferior, perioperative mortality was <1%. During progression into heart failure 28±2 days mortality in a cohort of 23 animals was 17% (n = 4). Out of the remaining 19 rats n = 6 sham rats and n = 6 ACF rats were used for the morphometric, hemodynamic, neurohumoral, and beta-adrenergic mRNA measurements. In addition, n = 2 sham rats and n = 5 ACF rats were used for electron microscopy.

**Table 1:** Morphometric data.

	Control (n = 6)	ACF (n = 6)
**BW (g)**	370±16 (330–419)	366±9 (345–395)
**Heart (mg)**	1410±49 (1270–1570)	2430±113 (2000–2730)*
**Heart/BW (mg/g BW)**	3.9±0.1 (3.5–4.1)	6.6±0.2 (5.7–7.0)*
**Lung (mg)**	1370±43 (1270–1490)	2675±204 (1930–3170)*
**Lung/BW (mg/g BW)**	3.8±0.2 (3.2–4.5)	7.3±0.6 (5.6–9.1)*
**Kidney (mg)**	1192±60 (1050–1340)	1160±62 (950–1330)
**Kidney/BW (mg/g BW)**	3.3±0.1 (3.1–3.5)	3.2±0.1 (2.8–3.4)
**Liver (mg)**	12824±877 (10620–15070)	13707±976 (10350–16500)
**Liver/BW (mg(g BW)**	35.0±1.6 (30.5–40.3)	37.4±2.4 (30.0–45.8)

Values are means ± SEM and range; n = 6 rats/group. BW, body weight.

* indicates a statistically significant difference betwenn groups (p<0.001).

### Systolic and Diastolic Dysfunction in ACF rats

**Figure 1:**
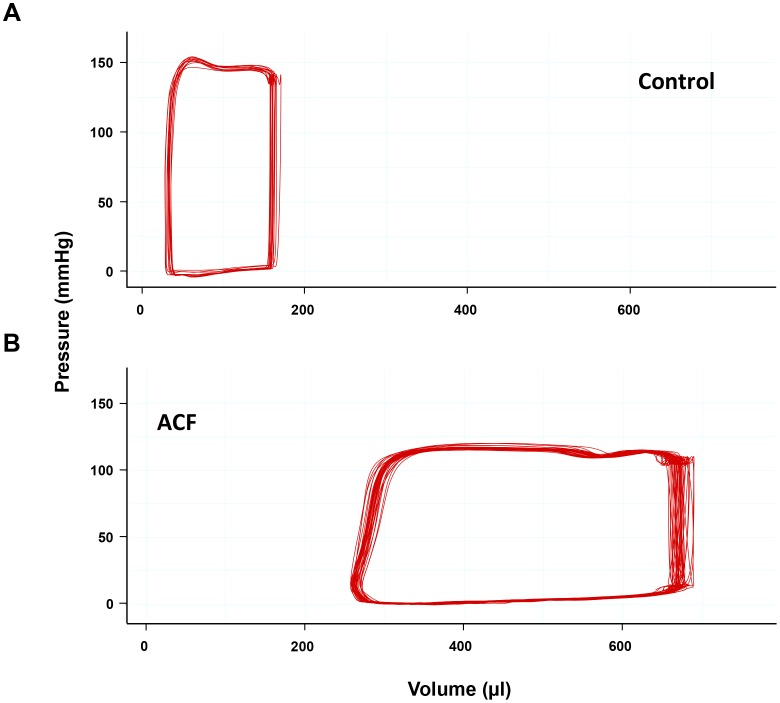
Representative pressure-volume loops of a sham-operated control rat (top) and an ACF rat (bottom).

Values for various hemodynamic parameters of the control and ACF groups are shown in Table 2. Central venous (CVP) and left end-diastolic pressure (LV-EDP) were significantly increased in ACF rats (p<0.01). Systolic and diastolic arterial blood pressures (SBP, DBP) were significantly reduced due to afterload reduction in rats with an aortocaval fistula (p<0.01). Cardiac output was nearly doubled in the ACF group. This was based on a nearly doubled stroke volume (p<0.01) forming a characteristic pressure-volume-loop in fistula-operated animals (Fig. 1 A and B). Left ventricular end-diastolic and end-systolic volumes (LVEDV, LVESV) were significantly higher than in the control group (p<0.01). However, left ventricular ejection fraction (LVEF) (p<0.01) and the maximum rate of pressure development (p<0.01) were significantly reduced. There was no overlap in the data distribution between a) ACF rats with increased heart and lung weight indices and decreased LVEF and b) control rats. Furthermore, a slowed cardiac relaxation became apparent as a reduced maximum rate of pressure decay (p<0.01) and prolonged time of Tau (p<0.01).

**Table 2:** Hemodynamic data.

	Control (n = 6)	ACF (n = 6)
**HR (min^−1^)**	386±13 (345–420)	345±14 (301–373)
**SBP (mmHg)**	155±7 (131–180)	122±6 (106–148)*
**DBP (mmHg)**	114±9 (72–134)	69±2 (63–76)*
**LVEDP (mmHg)**	5.0±0.3 (3.9–6.3)	13.9±1.3 (9.9–16.7)*
**CVP (mmHg)**	0.2±0.1 (0.0–0.7)	6.4±0.8 (3.6–8.4)*
**dP/dt max. (mmHg/s)**	15876±1128 (12143–18562)	9024±1207 (5616–13974)*
**dP/dt min. (mmHg/s)**	−9675±880 (−13344–−7715)	−5791±702 (−8714–−3614)*
**Tau (ms)**	8.0±0.4 (6.80–9.53)	13.4±1.1 (9.71–17.87)*
**Elastance (mmHg/µl)**	1.01±0.11 (0.68–1.32)	0.38±0.03 (0.31–0.48)*
**LVEDV (µl)**	188±8 (166–220)	633±51 (403–736)*
**LVESV (µl)**	48±2 (41–56)	400±38 (251–508)*
**SV (µl)**	140±7 (125–170)	233±24 (152–334)*
**CO (ml/min)**	54±4 (46–71)	81±10 (46–121)*
**LVEF (%)**	74±1 (72–77)	37±2 (30–45)*

Values are means ± SEM and range; n = 6 rats/group. BW, body weight.

* indicates a statistically significant difference between groups (*p*<0.05).

HR = heart rate; SBP = systolic blood pressure; DBP = diastolic blood pressure; LVEDP = left ventricular enddiastolic pressure; CVP = central venous pressure; dP/dt max. = maximum rate of pressure change of the left ventricle in systole; dP/dt min. = minimum rate of pressure change of the left ventricle in diastole; Tau = isovolumic relaxation constant; LVEDV = left ventricular enddiastolic volume; LVESV = left ventricular endsystolic volume; SV = stroke volume; CO = cardiac output; LVEF = left ventricular ejection fraction.

### Increased rBNP-45 plasma concentration in rats with heart failure

**Figure 2:**
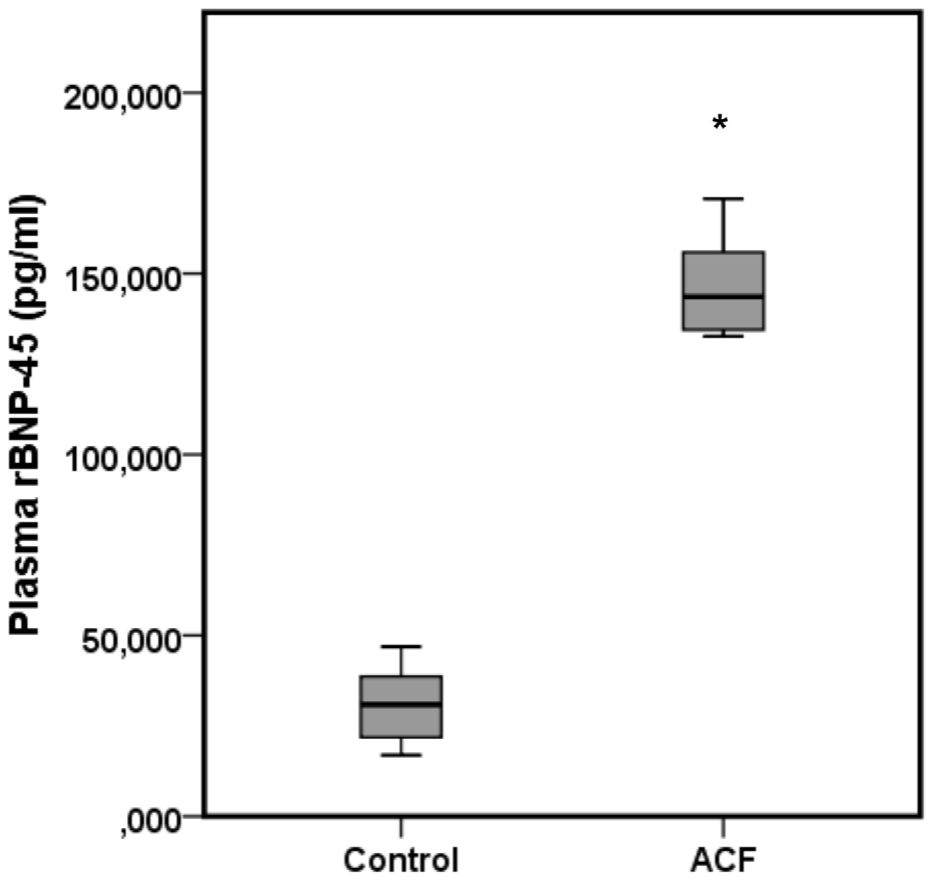
Total rBNP-45 plasma concentrations of control and ACF rats. There was no overlap in data distribution meaning ACF animals could be clearly distinguished from healthy animals. * indicates a statistically significant difference between both groups (p<0.01, n = 6 per group).

Values for the rBNP-45 plasma concentration from the control and ACF groups are shown in Figure 2. rBNP-45 plasma concentrations were significantly increased in the ACF group. Again, there was no overlap in data distribution between control rats and ACF rats.

### β1- and β2-AR mRNA downregulation in the LV

**Figure 3:**
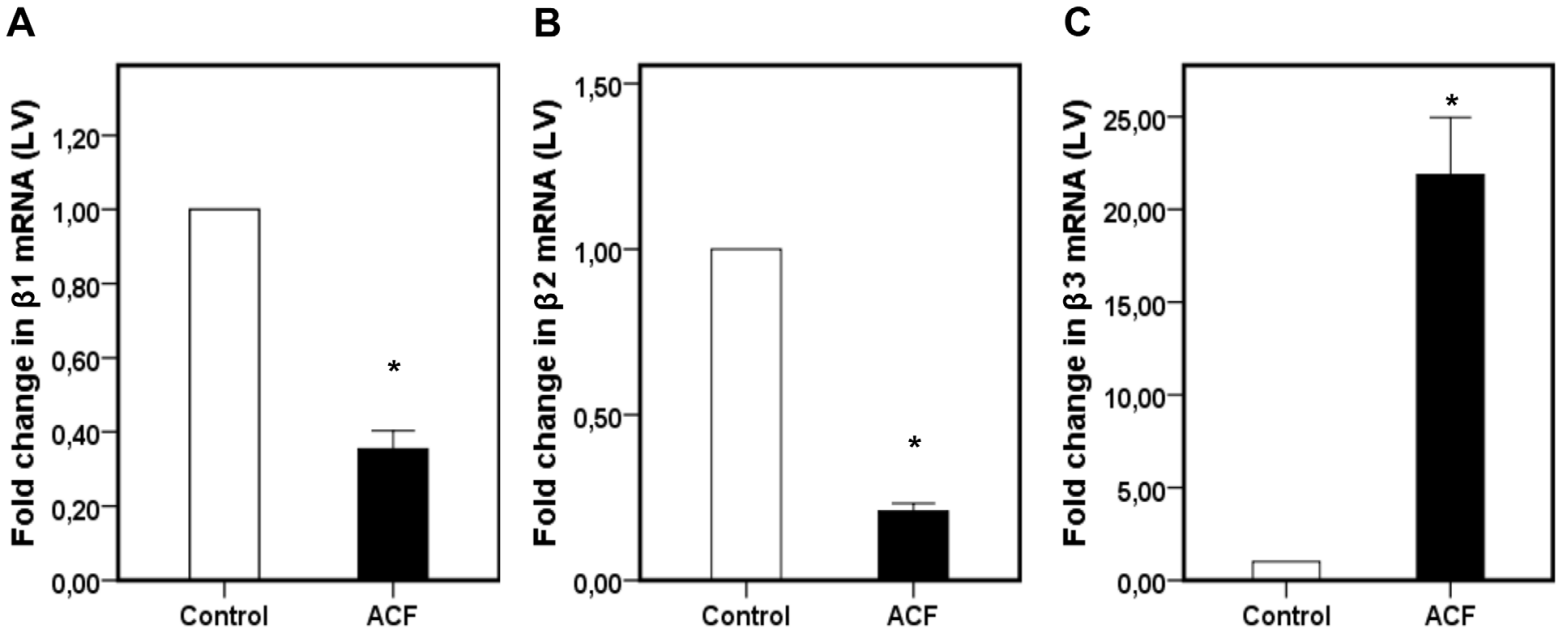
Fold change in β1- (A), β2- (B) and β3- (C) AR mRNA expression in the left ventricle of control and ACF rats. * indicates a statistically significant difference between both groups (p<0.01, n = 6 per group).

Values for the *β*1-, *β*2- and *β*3-adrenoreceptor mRNA from the control and fistula groups are shown in Figure 3. *β*1- and *β*2-adrenoreceptor mRNA expression was significantly decreased in the fistula group (p<0.01) (Fig. 3 A and B).

### β3-AR upregulation 28 days after shunt induction

In contrast to the significant downregulation of *β*1- and *β*2-adrenoreceptor mRNA in the left ventricle (LV) of rats 28±2 days after shunt induction, *β*3-adrenoreceptor mRNA of the LV was significantly upregulated in rats with an aortocaval fistula (p<0.01) (Fig. 3 C).

### Signs of progressive subcellular fragmentation in the myocardium of rats with an aortocaval fistula

**Figure 4:**
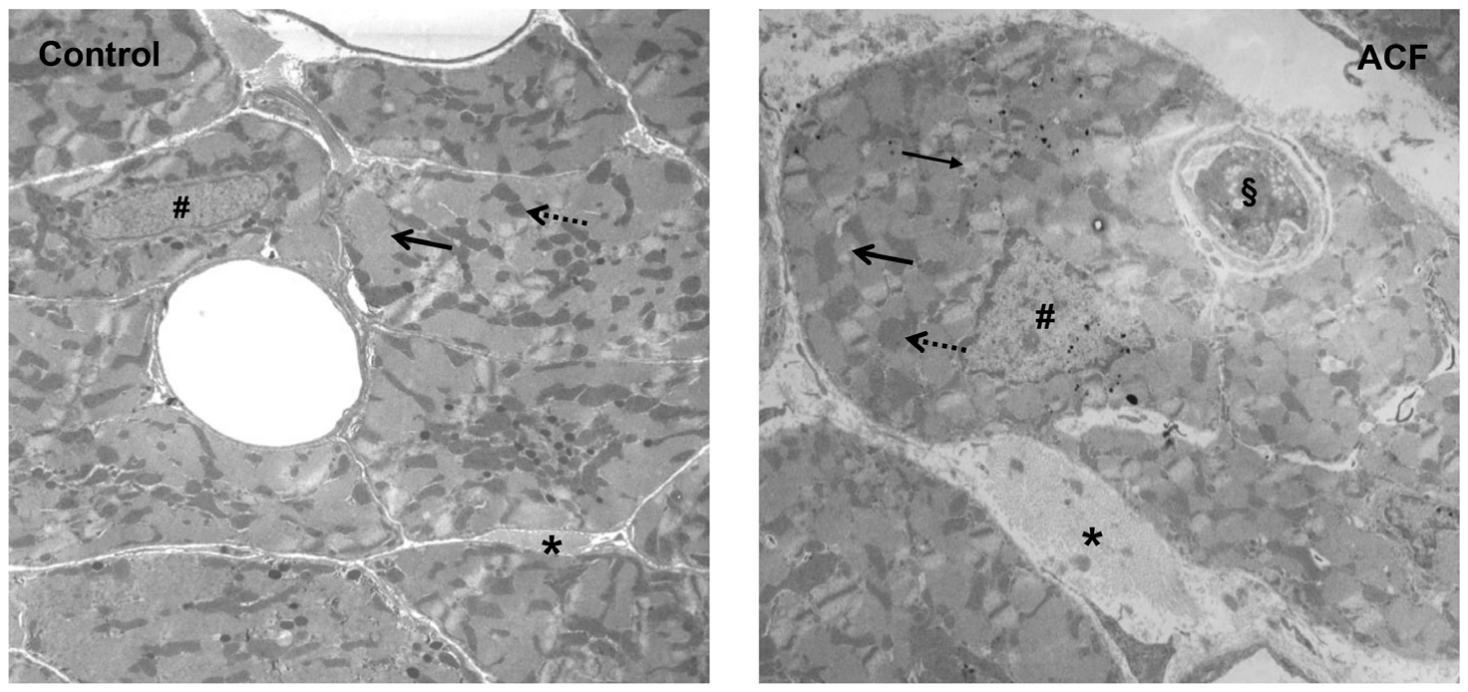
Electron microscopy in rats 4 weeks after induction of an aortocaval shunt (right) showing a) fragmented nuclear chromatin (#), b) reduced size of myofibrils ( ), c) swollen mitochrondria (←), d) vacuolization (→), e) dissolved cell-cell-contacts with degenerative appearances (*) and f) immigration of phagocytic leukocytes (§). Magnification: ×10.000.

A representative example of the changes of the subcellular myocardial structure is given in Figure 4. The electron microscopy image of the LV of rats in the ACF group showed: a) fragmented nuclear chromatin, b) reduced size of myofibrils, c) swollen mitochondria, d) vacuolization, e) dissolved cell-cell-contacts with degenerative appearances, and f) immigration of phagocytic leukocytes. This might suggest apoptotic changes in the left ventricle myocardium during the progressive course of heart failure.

## Discussion

In previous publications heart failure was inconsistent and started to develop earliest ≥8 weeks after fistula induction using a 18G-needle [4], [6]. Therefore, our goal was to modify the needle-technique to achieve a predictable CHF within a short time period to facilitate interventional studies with an improved assay sensitivity. In this context, we used a 16G instead of a 18G needle, which theoretically resulted in 69% larger cross-sectional area of the fistula inducing a more pronounced cardiac volume overload to hasten the pathophysiological processes. In animals of the 16G needle ACF group, our findings met criteria to characterize a severe CHF already after 28 days: 1) LV end-diastolic volume >550 µl [4], 2) lung mass >2500 mg [6], [17], 3) significant increase (>50%) in heart weight to body weight ratio compared with the control group and/or a heart weight index ≥5,0 mg/g BW [17], [18], and 4) elevated central venous pressure ≥4 mmHg [18]. As a new finding and in contrast to the previous studies using different fistula induction methods, e.g. microsurgery vs. different sized needles [4], [9], [19]–[21], these end points were reached within 4 weeks in every single rat of the here presented ACF group.

*In vivo* hemodynamic assessment of rats by means of a pressure-volume catheter has become the gold standard - besides echocardiography - in experimental models investigating adaptive changes due to myocardial infarction [22] or volume overload [23]. In using this modified approach, a complete hemodynamic characterization derived from a conductance catheter *in vivo* in this modified approach is shown for the first time. Intraventricular pressures and volumes in the control group were comparable to values previously published for healthy rats [10], [23], [24]. A nearly two-fold increase in the lung weight index accompanied by elevated central venous and left end-diastolic pressures denotes backward failure (“pulmonary congestion”). Finally, we demonstrated a clear separation between an increased heart and lung weight indices and a reduced LVEF in every ACF animal compared to every animal in the control group. This clearly depicts the transition from eccentric hypertrophy with preserved cardiac function to severe biventricular dilatation with decompensated heart failure due to pronounced volume overload in our modified approach [4], [7], [25], [26].

Today, progressive heart failure has been recognized to develop from a combination of genetic, neurohumoral, inflammatory [27], and biochemical factors. Therefore, biomarkers have become important for clinical risk stratification. Beside Troponin in the case of acute coronary syndrome [28], BNP is one of the most interesting biomarkers to grade the extent of heart failure to support medical decisions making and to monitor responses to therapy [29], [30]. An increase in BNP plasma concentration reliably reflects the neurohumoral activation due to persistent hemodynamic overload of the heart [31]. 28±2 days after fistula induction BNP plasma concentrations were significantly increased in our modified experimental model. This is consistent with previous results by Langenickel et al. demonstrating the superiority of cardiac BNP mRNA expression as a marker of the transition from compensated to overt heart failure in volume overloaded rats [17].

Also, subcellular fragmentation consistent with apoptotic changes of the myocardium has been recognized as an important pathophysiological mechanism promoting progression of heart failure [32]–[34]. This holds true for several causes of heart failure, including myocardial infarction [35], and pressure [36] and volume overload [37]. As an additional new finding in our modified experimental approach, we were able to demonstrate sites of severe subcellular fragmentation in cardiomyocytes of the left ventricle 28±2 days after fistula induction. This is extending previous findings which demonstrated a) increased apoptotic rate of the myocardium only 16 weeks after ACF induction using a 18G needle [37], and b) mainly in non-cardiomyocytes [38]. However, further studies have to confirm these data.

Abnormalities in myocardial catecholamine release and in β-adrenergic receptor (AR) density in patients with congestive heart failure have long been recognized [39], [40]. Adrenergic over-activity is one of the hallmarks of the heart failure syndrome and is associated with a poor prognosis [41]–[43]. As early as 1992, Hammond et al. reported blunted heart rate responsiveness to β1-AR stimulation in volume overloaded pigs due to β1-AR downregulation 5 weeks after fistula induction [44]. In failing human myocardium, β1-ARs are downregulated as studied with quantitative polymerase chain reactions in dilated and ischemic cardiomyopathy [45]. Also, Ihl-Vahl et al. were able to describe a decrease in mRNA-levels of beta 2-adrenergic receptors in the failing human heart [46]. In our results we demonstrate a β1- and β2-adrenoreceptor mRNA downregulation in the LV in rats with heart failure.

Almost two decades ago, the β3-adrenorecptor (β3-AR) has been identified in myocardial tissue [47]. In healthy myocardial tissue from rodents or humans, the mainly Gi-protein coupled β3-AR is only scarcely expressed. However, according to current knowledge [48]–[50], the β3-AR is upregulated in the failing heart of the ACF group which is consistent with our data from the ACF group.

In conclusion, our modified experimental model of heart failure using a 16G needle to induce an infrarenal aortocaval fistula has major advantages to investigate cardiac pathophysiology: congestive heart failure can reproducibly be induced within a relatively short and convenient time period. Combining morphometric, hemodynamic and biochemical parameters, the extent of heart failure can be well characterized. This modified model might facilitate the examination of various questions concerning CHF specifically by precisely timed interventions to determine pathophysiological pathways.
